# Euthanasia and assisted suicide in patients with personality disorders: a review of current practice and challenges

**DOI:** 10.1186/s40479-020-00131-9

**Published:** 2020-07-30

**Authors:** Lars Mehlum, Christian Schmahl, Ann Berens, Stephan Doering, Joost Hutsebaut, Andres Kaera, Ueli Kramer, Paul Anthony Moran, Babette Renneberg, Joaquim Soler Ribaudi, Sebastian Simonsen, Michaela Swales, Svenja Taubner, Ester di Giacomo

**Affiliations:** 1 National Centre for Suicide Research and Prevention, Institute of Clinical Medicine, University of Oslo, Oslo, Norway; 2 Department of Psychosomatic Medicine and Psychotherapy, Central Institute of Mental Health, Heidelberg University, Mannheim, Germany; 3 University Psychiatric Centre Duffel, CAPRI, faculty Medicine and Health Sciences, University Antwerp, Antwerp, Belgium; 4 Department of Psychoanalysis and Psychotherapy, Medical University of Vienna, Vienna, Austria; 5 De Viersprong Institute for Studies on Personality Disorders, Bergen op Zoom, The Netherlands; 6 Kanta-Häme Central Hospital, Hämeenlinna, Finland; 7 Department of Psychiatry, University of Lausanne, Lausanne, Switzerland; 8 Centre for Academic Mental Health, Department of Population Health Sciences, Bristol Medical School, University of Bristol, Bristol, UK; 9 Freie Universität, Berlin, Germany; 10 Department of Psychiatry and Legal Medicine, Hospital de la Santa Creu i Sant Pau, Autonomous University of Barcelona, UAB, Barcelona, Spain; 11 Centro de Investigación Biomédica en Red de Salud Mental, CIBERSAM, Madrid, Spain; 12 Stolpegaard Psychotherapy Centre, Copenhagen, Denmark; 13 NWCPP, School of Psychology, Bangor University, Bangor, UK; 14 Institute for Psychosocial Prevention, University-Hospital Heidelberg, Heidelberg, Germany; 15 School of Medicine and Surgery, University of Milan-Bicocca, Milan, Italy; 16 Psychiatric Department-ASST Monza, Monza, Italy

**Keywords:** Personality disorder, Euthanasia, Physician-assisted suicide

## Abstract

**Background:**

Over the last two decades an increasing number of countries have legalized euthanasia and physician-assisted suicide (EAS) leading to considerable debate over the inherent ethical dilemmas. Increasing numbers of people with personality disorders, faced with unbearable suffering, have requested and received assistance in terminating their lives. EAS in people with personality disorders has, however, received very sparse attention from clinicians and researchers. In this paper, we examine the literature on the practice and prevalence of EAS in people with personality disorders to date and discuss the associated challenges for research and practice.

**Methods:**

Narrative review of the literature combined with the authors’ collective experience and knowledge of personality disorders.

**Results:**

In six of the eight countries where EAS is currently legal, mental disorders are accepted as disorders for which EAS may be granted. In four of these countries, EAS in minors with mental disorders is also accepted. Our literature search resulted in 9 papers on the subject of EAS in people with personality disorders. These studies suggest that most clinicians who grant EAS have indeed perceived their patients’ suffering as chronic, unbearable and untreatable without prospect of improvement. The majority of patients with personality disorders had tried some form of psychotherapy, but very few had received any of the relevant evidence-based treatments. The decision to grant EAS based on a perception of the patient’s illness as being untreatable with no prospect of improvement, could, thus, in many cases fail to meet the due care criteria listed in EAS laws. People with personality disorders more often wish for death for extended periods of time than people without these disorders. However, there is ample empirical data to show that suicidal tendencies and behaviour can be treated and that they fluctuate rapidly over time.

**Conclusions:**

In light of our findings, we believe that the current legislation and practice of EAS for people with personality disorders is based on an inadequate understanding of underlying psychopathology and a lack of awareness about the contemporary treatment literature. Moreover, we assert that this practice neglects the individual’s potential for having a life worth living.

## Background

On February 20th 2020 Portugal’s parliament provisionally approved a bill to legalize euthanasia and physician-assisted suicide (in the following collectively labelled EAS), thus joining a burgeoning group of nations who have chosen to permit what has for many centuries, remained a prohibited and morally condemned practice. Similar legislative changes are currently taking place in other countries. Invariably, what motivates legislators to decriminalize EAS, is to end what is considered as unbearable, untreatable and unnecessary suffering in people with incurable illness and, thus, assisting a peaceful death. Although somatic suffering is almost always the main focus of law-makers attention, life-ending assistance for people with mental illness has become more common in recent years. Over the past decade, we have identified the fact that an increasing number of people with personality disorders (PD) have requested and received EAS. Nevertheless, EAS in this group has received very sparse attention. In this paper, we examine the literature on the practice and prevalence of EAS in people with PD to date, discuss associated challenges and provide recommendations for policy makers, clinicians and researchers.

### A brief history of euthanasia

The Greek term euthanasia (εὐθανασία) denotes a good (*eu*) death (*thanatos*) and was from antiquity originally not associated with physician-assisted dying, but regarded as a natural and highly desirable course at the end of one’s life [1]. Indeed, under the Hippocratic oath, any kind of assisted dying is explicitly forbidden [2]. In the medieval period and early modern age, euthanasia was generally prohibited and it was only at the beginning of the sixteenth century that so-called “mercy killing” was openly discussed and supported by some philosophers and ethicists. Euthanasia was regarded as a facilitation of dying, assisted by physicians and potentially, but not necessarily, shortening life [1]. In the late 19th and early 20th centuries, on the background of Darwinism and the Eugenics Movement, euthanasia became more frequently discussed and practiced, culminating in the ideology and practices embraced by the Nazi regime in Germany, where so-called “Gnadentod” (German for “mercy death”) became a euphemistic term for the coordinated killing of mentally ill and physically or mentally disabled people who did not fit into the prevailing ideal of human beings. As a consequence, in German-speaking countries the term “euthanasia” is still negatively charged and has, thus, been banned from the societal discourse and replaced by the term “aktive Sterbehilfe”, which is close to the English term “assisted dying”. This linguistic situation is different in many other countries in the world, where euthanasia in the public eye seems to represent a more or less desirable facilitation of death without pain or other suffering.

A contemporary definition of euthanasia is proposed by Denys [3]: “Euthanasia means that the physician acts directly to end the patient’s life, e.g., by giving a lethal injection”. In contrast “Physician-assisted Suicide” (PAS) is the situation in which a physician provides the specific means and instructions to a patient with the intention of ending the patient’s life, but where the patient him/herself performs the act of ending his or her life [4]. In 1942, Switzerland was the first country to legalize PAS but not euthanasia. However, according to Swiss legal practice, the latter will not be punished, if it is delivered free of selfish motives [5]. After 2001, seven more countries have legalized PAS and/or euthanasia (for an overview see Table 1). The Netherlands, Belgium, Luxembourg as well as Colombia and Canada now all permit euthanasia, whereas Switzerland, parts of Australia (the state of Victoria), and ten states of the USA only legalized PAS. In addition, the legislature of New Zealand has just recently allowed euthanasia, but the final decision will be made in a referendum in 2020. In Germany the Federal Constitutional Court (Germany’s highest court) in February 2020 overturned a ban on organized assisted suicide, thus declaring that the right to die includes the freedom to rely on the voluntary help of another person. In all of the countries having legalized PAS and/or euthanasia, so far, more or less detailed procedures have been described as requirements for directly or indirectly helping people to die. Usually, an examination by a medical doctor has to take place, and the person has to be able to decide freely without being influenced by any relevant cognitive impairment or external pressure.

**Table 1:** Overview of the legislation on euthanasia and physician-assisted suicide in the different countries where it is established

Country	Netherlands	Belgium	Luxemburg	Switzerland	Colombia	Canada	AustraliaVictoria	USAOregon, Washington, Montana, Vermont, California, District of Columbia, Colorado, New Jersey, Maine, Hawaii
Entry into force of law	2001	2002	2009	1942	2015	2016	2017	2008–2019
Physician-assisted suicide (PAS)	+	+	+	+	+	+	+	+
Euthanasia (EUT)	+	+	+	–	+	+	–	–
Allowed to be given to mentally ill	PAS/EUT	PAS/EUT	PAS/EUT	PAS	–	PAS/EUT	–	PAS
Age limit	≥12	no limit	≥18	No regulation, but usually only applied ≥18	≥6	≥18	≥18	≥18
Reference	https://english.euthanasiecommissie.nl/	http://www.ethical-perspectives.be/viewpic.php?LAN=E&TABLE=EP&ID=59 http://www.ejustice.just.fgov.be/mopdf/2014/03/12_1.pdf#Page67 (Dutch and French language)	https://guichet.public.lu/de/citoyens/famille/euthanasie-soins-palliatifs/fin-de-vie/euthanasie-assistance-suicide.html (German language) http://legilux.public.lu/eli/etat/leg/loi/2009/03/16/n2/jo (French language)	https://www.admin.ch/opc/en/classified-compilation/19370083/index.html https://www.bj.admin.ch/bj/en/home/gesellschaft/gesetzgebung/archiv/sterbehilfe/formen.html	https://www.minsalud.gov.co/Normatividad_Nuevo/Resolución%201216%20de%202015.pdf (Spanish language) López- Benavides 2018	https://www.canada.ca/en/health-canada/services/medical-assistance-dying.html	https://end-of-life.qut.edu.au/euthanasia https://www2.health.vic.gov.au/hospitals-and-health-services/patient-care/end-of-life-care/voluntary-assisted-dying	www.deathwithdignity.org

Although PAS or euthanasia were originally intended to facilitate death without pain or other suffering from physical illness, people with mental illness have gradually also been considered eligible.

## Methods

This paper is based on a narrative review of the literature combined with the authors’ collective experience and knowledge of personality disorders from extensive clinical practice, clinical research and treatment development, and involvement in the formulation of national policy in relation to the management of personality disorder.

### Literature search

We searched the Medline database (OVID) for peer reviewed articles and letters to the Editor published in English through October, 2019 with the following MeSH-terms: “Euthanasia”, “Euthanasia, Active”, “Euthanasia, Active, Voluntary”, “Suicide, Assisted”, “Personality Disorders” and “Borderline Personality Disorder”. No language or time filters were applied. The literature search resulted in 9 papers; 2 regular articles based on empirical studies, 4 literature reviews and 3 comments/letters to the editor. In the following, we summarise the main findings and conclusions from these publications.

## Results

Table 1 provides an overview of the legislation on euthanasia and physician-assisted suicide in the different countries where it is established. The majority of the countries allowing EAS restrict it to people above the age of 18 years, whereas the Netherlands offer both PAS and euthanasia from the age of 12 years, Colombia permits PAS in children after the age of 5 years, and Belgium has abolished any age limit (Table 1).

In the case of Belgium, the law requires a repeated wish from the child himself and permission from parents (except in the case of so-called ‘emancipated minors’ where a minor, through court order or other means, legally becomes an adult). Few cases of EAS in minors have so far been reported; all in cases of physical illness. Some countries allow EAS in mentally ill people, provided they are considered able to express their free will (this is not required by Colombia and Australia, but here EAS is not permitted for psychiatric reasons). To be granted euthanasia in Belgium, to date the country with the most liberal EAS legislation, a person must be “in a medically futile condition of unbearable and untreatable physical or psychological suffering, resulting from a serious and incurable disorder caused by accident or illness” [6]. The Federal Control and Evaluation Committee on Euthanasia in Belgium reports [7] that about of 3% of EAS-cases are mainly linked to mental disorders. Thienpont and colleagues [8] conducted a retrospective analysis of medical records of the first 100 consecutive patients (23 men and 77 women) who requested euthanasia for psychological suffering associated with mental disorders in the years between 2007 and 2011. The most common of these disorders were treatment resistant mood disorders (*n* = 58) and personality disorders (*n* = 50), whereas 29 patients had both types of disorder. Among the 50 patients with a PD, 27 had borderline PD (BPD), 3 had dependent PD, 2 had histrionic PD and 18 had some other PD or PD not otherwise specified. In all patients with PD suffering was reported to be chronic, constant and unbearable, “without prospects of improvement, due to treatment resistance” [8]. The study does not specify or operationalize the concept of “treatment resistance” or how it was measured. In total, 48 of all of the 100 euthanasia requests were accepted, of which 35 were carried out, but the authors do not specify how many of these had a PD diagnosis.

In 2018, 4% of all deaths in the Netherlands were due to EAS, and among all cases of EAS (*N* = 6126) around 1% involved patients with a mental disorder according to the Dutch regional euthanasia review committees (RTE) [9]. Since 2013 the RTE has published all psychiatric cases of EAS on their website. Kim and co-workers reviewed the first 66 psychiatric cases of EAS published and found that 52% of these were patients with personality disorders or difficulties [10]. The prevalence of PD was higher in patients who were younger. Recently, a larger Dutch study based on content analysis was published by Nicolini et al. [11]. Among the 116 cases published by the RTE, a diagnosis of PD was likely, according to the authors’ clinical judgement, in just under two-thirds of cases (*n* = 74), although a PD diagnosis had been explicitly made by the patients’ doctors in only 48 (41%) cases. The majority (72%) of patients with PD had received some form of psychotherapy, but mostly of unspecific nature, and only one patient (1%) had received any of the standard PD-specific evidence-based treatments currently in existence.

Most of the people who request EAS for mental illness appear to be experiencing multiple mental health problems. In the study by Kim et al. [10] depressive disorders (55%), posttraumatic stress disorder and other anxiety disorders (42%) were prominent whereas 52% of the patients had personality-related problems, sometimes without a formal PD diagnosis, more so in younger patients. Social isolation or loneliness were mentioned in 56% of the cases.

## Discussion

Our review of the literature suggests that a large proportion of people with mental disorders who request and receive EAS are people with personality disorders. It is difficult to reach firm conclusions about prevalence figures and proportions based on the limited number of studies and cases reported in each study. Yet, we think these findings make for disturbing reading, particularly in light of the time-sensitive changes that can occur in the psychopathology of personality disorder. We consider this issue further below.

### Personality disorders and the wish for death

Suicidal and self-harming behaviours are frequently seen in people with personality disorders, in particular borderline personality disorder (BPD), where this is one of the diagnostic criteria [12]. Emotional and behavioural dysregulation is characteristic of BPD and is closely linked to suicidal behaviour [13]. In addition, suicidal behaviour in people with BPD is often linked to the wish to seek help, to communicate or to solve interpersonal problems. An important aspect of BPD individuals’ problems is their difficulty with regulating their relationships with other people maintaining nurturing close interpersonal relationships over time [14]. This extends to the clinical setting where patients with BPD all too often feel disappointed, rejected or invalidated by their therapists and thus terminate treatment at an early stage [15].

A wish for death and an increased risk of suicide may be prominent, although less well documented, among people with other personality disorders. In narcissistic personality disorder, there is frequently a hypersensitivity to the evaluations of others and a fluctuation in the self-esteem between grandiose and depleted states, depending on life circumstances [16, 17]; these are personality features associated with an increased risk of both suicidal and non-suicidal self-injury [18, 19]. Suicidal behaviour is frequently seen in people with antisocial personality disorder, where it is often associated with severe problems in interpersonal relations and with the justice system.

It follows from the above that people with personality disorders on average more often contemplate death and may have a stronger wish for death for more extended periods of time than is the case for people without these disorders. We have, however, no data to suggest that these tendencies of thinking about suicide or wishing for suicide are completely immutable to change. Rather they are very likely learnt dysfunctional behaviours, and they fluctuate rapidly. An individual who frequently experiences strong and painful feelings of helplessness, sadness and entrapment may find that thinking of death and suicide will offer some sense of control (“I can stop the pain”) and relief. Thus, it will be likely that this person will be more inclined to think about suicide next time the painful feelings become overwhelming. This reinforced pattern of frequently thinking of and/or wishing for death and suicide is very familiar to many people with BPD and other personality disorders [20], but it may seem difficult to accept or to understand for other people, including health-care personnel. In turn, this frequently leads to a host of dysfunctional transactions between people with severe PDs and their families and carers.

Under the principle of self-determination, it can be argued that only patients themselves can be the judge of what is best for them and that it should not be up to doctors to make interpretations of a patient’s wish for death. Whereas we respect this principle, it is still the duty of clinicians to provide due care and use their knowledge to act in accordance with what they believe is in the best interest of their patient. This is also emphasized in the due care criteria in several of the current laws and guidelines for EAS, for example in the Dutch guideline where it mentions explicitly that if the wish for death is a symptom of a mental disorder, it should be treated and not lead to EAS.

We believe that in contexts where EAS for people with PDs is available, particularly precarious circumstances are present, with the potential for adverse consequences. Frequent suicidal communications and requests for EAS may lead carers to underestimate the basic criterion for granting such requests; that the patient’s situation must entail unbearable suffering with no prospect of improvement and no alternative to end the suffering. However, in most cases even severe suicidal tendencies and self-harming behaviour can be treated and individuals can be helped to recover. We must emphasize that a range of psychosocial interventions, including cognitive-behavioural therapy, dialectical behaviour therapy and mentalization based therapy have all been shown to reduce suicidal and self-harming behaviour in randomized trials with both adolescents [21] and adults [22]. Admittedly, even though effects of specialized treatments are good for the average patient, some patients are non-responders or even deteriorate or drop out early. We would argue that, in cases of non-response, patients with PD should be offered an alternative evidence-based treatment before EAS is considered. Currently, we don’t know how large proportion of patients with PD requesting euthanasia could have been treated effectively, and how big the group is who would not respond to even the best of treatments. There is a great need for more studies to shed light over these important questions.

### The transactional aspects of asking for and granting of EAS in patients with PD

A request for EAS from a severely ill patient, in reality, may be a request for communication about loneliness or mental suffering, or an attempt by the patient to find a reason to continue living through eliciting a dialogue with his/her doctor or mental health worker [23]. Studies have shown, however, that people with BPD have significant difficulties with correctly appraising other people’s emotions and intentions through observing their facial expressions [24]. They frequently associate neutral faces as expressions of sadness, aggression or disgust. Such difficulties could result in patients believing that their physicians agree with them that they would be better off dead. To our knowledge, there are no empirical studies of whether, and how frequently, such misperceptions occur in the context of requests for EAS and whether they have a significant influence on the outcome of these requests. In our experience, this is just one of several possible mechanisms through which patients and their carers might severely miscommunicate with respect to requests for and granting of EAS.

Authors writing about EAS from a psychodynamic perspective have highlighted that a request for EAS may be viewed as an expression of rage at the physician for a variety of reasons – for example, appearing to give up (or indeed giving up) on the patient, or not provide a cure [25]. Clinicians often find it hard to work with these clients because being close to someone who is in unbearable emotional pain is difficult. Furthermore, clinicians frequently experience their transaction with patients with BPD as more difficult and demanding than with other patients. This is very accurately described by the well-known American psychiatrist Allen Frances as follows: “Most of us have a much greater immediate empathy for a patient’s depression or anxiety, and even for violent impulses and psychotic thinking, than we do for the relief some patients feel when they hurt and scar themselves. The typical clinician (myself included) treating a patient who self-mutilates is often left feeling some combination of helpless, horrified, guilty, furious, betrayed, disgusted and sad” [26].

Again, it is important to remember that our knowledge on what influences physicians’ decisions when dealing with requests for EAS is very limited due to a severe lack of research in this field. However, in a Dutch study, a lower proportion of physicians found it conceivable that they would grant EAS in patients with mental illnesses (34%) than the proportion who found it conceivable that they would grant EAS in patients with physical illnesses, such as cancer (85%) [27]. Interestingly, general practitioners were 2.6 times more likely than clinical specialists to find it conceivable that they would grant EAS in patients with mental illnesses. This could mean that with higher level of clinical expertise may follow a reduced willingness to grant EAS for mental illnesses.

Patients with BPD and suicidal and self-harming behaviours are typically regarded as being hard to treat. This seems to have been the case with patients with PD in the study of Thienpont et al. [8] who were perceived as being “untreatable”. However, the question whether patients with PD are untreatable or “uncurable”, as demanded by Belgian law, is debatable. The most prevalent PD diagnosis in this particular study was Borderline PD, which although often a long-term condition, is not, contrary to popular belief, an incurable disorder. Over the past twenty years, treatment studies have flourished showing that a diverse range of manualized treatments for BPD such as dialectical behaviour therapy (DBT), transference-focused psychotherapy (TFP), mentalization based therapy (MBT), or schema therapy are effective with medium to large effect sizes and remission achievable in a high percentage of cases [28, 29]. National guidelines (e.g. in Germany or the UK) recommend one of the evidence-based psychotherapies mentioned above as first-line treatment [30]. They also stress the importance of careful delivery and supervision of these treatments by trained therapists, and the potential benefit of adjunctive psychotropic medication for symptom relief. Currently, there is a scarcity of research into the question of whether patients with PD who requested EAS were offered evidence-based PD-specific treatment. We think, however, that it is doubtful whether these guidelines have been followed in the majority of individuals with BPD who have requested EAS. We strongly recommend that adherence to guidelines for state-of-the art treatment for PDs, and BPD in particular, are included in the decision-making process underpinning EAS applications.

## Conclusions and recommendations

Based on the scant published literature, we have serious concerns about the practice emerging in an increasing number of countries of facilitating EAS for people with personality disorders. This appears to be based on faulty assumptions about the underlying psychopathology and a lack of awareness about the contemporary treatment literature, particularly on borderline personality disorder. First, we would argue that wishes for death or suicide, even when clearly articulated by the patient to doctors or next-of-kin, and even if it represents the true will at that very moment, this desire or wish for death will likely change in many of these cases. As we have pointed out above, such an articulated death wish, can be a symptom of the disorder and may in reality convey several other possible messages, that have more to do with the patient feeling abandoned, disappointed or angry. It may also convey a wish for help to live rather than a wish for help to die. Second, we would claim that the notion of personality disorders as “untreatable” conditions and “without prospects of improvement” are based on outdated knowledge about the state of PD treatment. Today, a range of effective psychotherapeutic interventions are available for people with personality disorders in most of the countries that have so far legalized EAS. That this has seemingly escaped the attention of both legislators and expert medical communities is deeply disturbing. It may be that the current lack of effective psychotropic medication to treat personality disorders could have made many physicians and psychiatrists not specializing in PD treatment less optimistic about the prognosis in people with PDs and the prospects of receiving effective treatment in general. We urgently call for a revision of the current legislation and practice of EAS for people with personality disorders which we believe, is currently based on an inadequate understanding of these peoples’ needs and their potentials for having a life worth living.

We recommend that professionals involved in making decisions regarding granting of EAS as a minimum should receive training covering a) insight in the fluctuating nature of suicidal thinking and its emotion regulation function, b) the evidence that PD – but especially BPD – is treatable, c) the risk of miscommunication around this issue given the impairments in identifying / reading emotions in others combined with a sense of being a burden to others and d) an examination of their own attitudes and beliefs towards suicidal and self-harm behaviour with particular attention to their own emotional responses. We further recommend that EAS Guidance should require that clients with BPD should have had a substantial period of treatment in at least two of the evidence-based approaches from an appropriately trained and supervised clinical team which has led to no noticeable improvement before EAS can be considered. Given the high likelihood of change in presentation of any mental disorder in minors, EAS should not be considered on the grounds of any established or emerging mental disorder for those under 18 years. Finally, we recommend legislators involved in EAS legislation and policy making should make provisions for funding of research into the current practice of EAS in people with mental disorders.

## Data Availability

Not applicable.

## Abbreviations

EAS: Euthanasia and physician-assisted suicide
PAS: Physician assisted suicide
PD: Personality disorder
BPD: Borderline personality disorder
